# Anticipatory and Compensatory Postural Adjustments in Response to External Lateral Shoulder Perturbations in Subjects with Parkinson’s Disease

**DOI:** 10.1371/journal.pone.0155012

**Published:** 2016-05-06

**Authors:** Alexandre Kretzer e Castro de Azevedo, Renato Claudino, Josilene Souza Conceição, Alessandra Swarowsky, Márcio José dos Santos

**Affiliations:** 1 Department of Physical Education, Center of Health and Sport Sciences, Master in Human Movement Sciences program, Santa Catarina State University, Florianópolis, Santa Catarina, Brazil; 2 Department of Physical Therapy, Center of Health and Sport Sciences, Master in Physical Therapy program, Santa Catarina State University, Florianópolis, Santa Catarina, Brazil; 3 Department of Physical Therapy and Rehabilitation Science, School of Health Professions, University of Kansas Medical Center, Kansas City, Kansas, United States of America; Duke University, UNITED STATES

## Abstract

The purpose of this study was to investigate the anticipatory (APA) and compensatory (CPA) postural adjustments in individuals with Parkinson’s disease (PD) during lateral instability of posture. Twenty-six subjects (13 individuals with PD and 13 healthy matched controls) were exposed to predictable lateral postural perturbations. The electromyographic (EMG) activity of the lateral muscles and the displacement of the center of pressure (COP) were recorded during four time intervals that are typical for postural adjustments, i.e., immediately before (APA1, APA2) and after (CPA1 and CPA2) the postural disturbances. The magnitude of the activity of the lateral muscles in the group with PD was lower only during the CPA time intervals and not during the anticipatory adjustments (APAs). Despite this finding, subjects with PD exhibit smaller COP excursions before and after the disturbance, probably due to lack of flexibility and proprioceptive impairments. The results of this study suggest that postural instability in subjects with PD can be partially explained by decreased postural sway, before and after perturbations, and reduced muscular activity after body disturbances. Our findings can motivate new studies to investigate therapeutic interventions that optimize the use of postural adjustment strategies in subjects with PD.

## Introduction

Parkinson's disease (PD) is one of the most prevalent neurodegenerative diseases affecting 1.8% of the elderly population (those over 65 years old) [1]. It is estimated that the prevalence of this disease in 2020 will be 3.3% of the elderly population [2]. PD is characterized by a progressive degeneration of the dopaminergic neurons of the substantia nigra pars compact in the mesencephalon, which is part of the basal ganglia and specific site of dopamine production [3]. The degenerative changes create an excessive inhibition pattern that generate the following cardinal symptoms in subjects with PD: resting tremor, bradykinesia, rigidity and loss of postural reflexes [4]. These symptoms contribute to severe postural instability and a high rate of falls observed in this population [5, 6].

Falls caused by postural disturbances are minimized or prevented by efficiently using the strategies of postural adjustment, which are essential for body equilibrium in response to perturbations of posture. These strategies include anticipatory postural adjustment (APA) and compensatory postural adjustment (CPA). The former involves muscle responses or small body shifts that occur before postural disturbances, and its main role is to minimize postural perturbations that are about to occur [7]. APA is based on a subject’s previous experiences and learning [7]. The other type of postural adjustment, CPA, is associated with muscle activity and body movements after postural perturbations; hence, they serve to minimize the destabilizing effects caused by disturbance of posture [8]. CPA can be manifested following both predictable and unpredictable perturbations [9]. It has been shown that the magnitude of both APA and CPA depends on, but is not limited to, the direction [10, 11] and degree of postural disturbances [12] which are classified as either internal (generated by forces and torques developed during the subject’s movement) or external (produced by forces around them).

During lateral perturbations of posture, the central nervous system (CNS) coordinates muscle activity bilaterally in order to minimize the postural disturbance [13, 14]. For instance, when healthy subjects were exposed to perturbations induced in the lateral and oblique directions, in a relatively stable posture (feet shoulder-width apart), a pattern of co-activation was observed between right and left external oblique (EO) and gluteus medius (GM) muscles [11]. In contrast, during similar perturbations in the anteroposterior direction, a pattern of reciprocal activation was observed between ventral (rectus femoris and rectus abdominis) and dorsal (biceps femoris and erector spine) muscles, i.e., activation of ventral and inhibition of dorsal muscles. The authors suggest that co-activation patterns of these lateral muscle pairs during postural disturbances in the lateral direction might be due to differences in body biomechanics between frontal and sagittal planes. In bipedal support, movements to maintain postural sway in the sagittal plane occur at ankle, knee and hip joints, individually or simultaneously, within higher amplitudes [15], which facilitates postural adjustments in response to perturbations in an anteroposterior direction. In contrast, these joints are more restricted in the frontal plane; hence, motion in one joint may cause a higher change in others. This condition limits the capability to respond to postural perturbations in a lateral direction, particularly the ones involving feet-in-place responses (when there was no change in the position of the participants’ feet). Thus lateral muscles of leg and trunk are co-activated for greater postural stability [16, 17]. To date, there is no data about these muscle activities in subjects with PD during similar postural disturbances.

Studies have shown that subjects with PD show a decrease in the magnitude of muscle activity during the strategies of postural adjustments with subsequent changes in postural balance [18–20]. For example, they showed a decrease in the magnitude of EMG activity in APAs during shoulder and elbow flexion [18, 20] and in CPAs in response to sudden platform translations [19]. Furthermore, studies have shown that subjects with PD reacted more slowly (delayed activation of postural muscles) during CPAs when perturbed [21]. In addition, they exhibited reduced center of pressure (COP) displacement during postural disturbances induced either through a movable force platform [22] or during voluntary movement [23]. Although these studies provide important information on the strategies of postural adjustments in subjects with PD, it is still unknown how these individuals use APAs followed by CPAs in terms of muscle activity and postural sway in response to lateral perturbations. Lateral instability of posture is associated with past falls [24] and future risk of falls in older individuals [25], which are frequently associated with hip fractures in this population [26].

The principal aim of this study was to investigate the APAs followed by CPAs in subjects with PD during external lateral disturbance of posture. Participants with PD and a matched control group received lateral disturbances at the shoulder level while the EMG activity of selected postural muscles and the lateral COP displacement (average) were recorded and analyzed. Based on previous studies, we hypothesized that the group with PD will show a decrease in the magnitude of postural muscle activity during the APAs and CPAs when compared to the control group. In addition, because of the characteristics of the disease (rigidity and bradykinesia), subjects with PD might exhibit a smaller excursion of the COP after the disturbances (CPAs). A better understanding of APAs and CPAs in subjects with PD may assist in developing new protocols for exercises and therapies to improve postural stability and reduce the risk of falls in this population.

## Method

### Ethics Statement

The study protocol was approved by the Ethics Committee in Research in Human Beings (*CEPSH*; initials in Portuguese language) of the Santa Catarina State University (protocol number 172/10). All participants were informed about the objectives and procedures performed in the research and had the capability to consent and sign a written informed consent.

### Subjects

A total of 26 adults participated in this research. They were divided into two groups: 13 participants (8 males and 5 females) with PD (PD group) and 13 healthy subjects (control group), matched by age, gender and physical characteristics (height and weight). All the participants were recruited in the University campus, Center of Health Sciences and Sports, and were in “on” medication phase (dopamine intake—action period). Both healthy and PD participants in this study had moderate levels of physical activity, according to the International Physical Activity Questionnaire (IPAQ), which was validated for Brazilian older populations [27]. The IPAQ has been shown to be an appropriate tool for quantifying activity levels in subjects with PD, as well [28, 29]. A moderate level is equivalent to 150 minutes of physical (running, swimming, etc.) or recreational (volleyball, dancing, etc.) physical activity per week. The participants with PD were involved in a physical therapy program at the University’s clinic twice a week. The program activities included stationary biking, walking on a treadmill, going up and down stairs, weight lifting exercises, and proprioceptive and gait training. The healthy controls were engaged in regular programs of supervised physical activity (one hour twice a week) for the community at the University. The program included, but was not limited to, swimming, walking, weight lifting, dancing, and hydro-gymnastics.

The inclusion criteria for participants with PD were the following: a) clinical diagnosis of the disease by a physician; b) early- (1 to 2) or mild- to moderate-stage (2 to 3) of the disease, according to the modified Hoehn Yahr scale [30], which encompasses the following stages: 1.0: unilateral involvement only; 1.5: unilateral and axial involvement; 2.0: bilateral involvement without impairment of balance; 2.5: mild bilateral disease with recovery on pull test; and 3.0: mild to moderate bilateral disease; some postural instability; physically independent; c) good cognition based on the Mini Mental State Examination (MMSE), and d) willingness to participate in the study. The exclusion criteria for the experimental group were the following: a) Parkinson's disease in advanced stage, i.e., > 3 according to the modified Hoehn Yahr scale [30], and b) orthopedic, rheumatic, vestibular, auditory, or severe visual impairment that could prevent the task performance. All participants in the control group were in good health, with no cognitive impairments and diseases related to the neurological, musculoskeletal or vestibular systems that would interfere with the performance of the task.

### Instruments and Materials

The participants filled out an identification form including age (in years), gender (male or female), and time of the PD onset (years), and side dominance. They also answered the Unified Parkinson's Disease Rating Scale (UPDRS), which provides a global assessment of Parkinson’s disease (Part I: Mentation, Behavior and Mood; Part II: Activities of Daily Living; Part III: Motor; Part IV: Complications) and serves to monitor its progression [31]. The four parts of the UPDRS included 42 items concerning symptoms and disabilities each ranging from 0 (absent) to 4 (present most of the time), totaling a maximum score of 168 points. The higher the scores the greater the disease severity. In addition, the subjects with PD were classified according to the disease staging established by the Modified Hoehn and Yahr Scale [30]. This scale is based on the aforementioned subjects’ impairments. To assess the cognitive function, all study participants took the MMSE test following the cutoff scores established by Bertolucci et al. [32], which were based on the age and educational level of the Brazilian population. In this study, the normal cognitive function cutoff score for illiterate subjects is 13, for those with elementary and high school 18, and for subjects with higher education the cutoff score is 26, irrespective of age.

A force platform (AMTI-OR 6–7, Watertown, EUA^®^) positioned on the floor was used to register the ground reaction forces and the associated moments. The EMG of the muscles was evaluated via electromyography (EMG System of Brazil ^®^, model 811C, São José dos Pinhais, SP, Brazil), with an analog output, a gain of 2000, a band pass filter from 23 to 500Hz, a Common Mode Rejection Ratio (CMRR) greater than 80 dB, and a 16-bits differential amplifier. Timing of the perturbation was recorded by an accelerometer (EMG System do Brasil^®^, model ACL13000/03, São José dos Pinhais, SP, Brazil) attached to the pendulum that was used to generate the postural perturbations (see below). All signals were sent to a computer through a digital/analog acquisition system (model PCI 6259, Austin, USA), with a frequency of 1000 Hz and resolution of 16 bits, acquired in a LabView Signal Express environment (National Instruments^®^, Version 4.0. 0, 2010 Austin, USA).

### Procedures

Disposable surface electrodes (3M ^®^, 223BRQ, Sumaré, SP, Brazil) were placed on the skin of individuals within an inter-electrode distance of 20mm, after cleansing with 70% alcohol and shaving the area, when necessary. The EMG electrodes were placed bilaterally over the following muscles: right (rGM) and left (lGM) gluteus medius, right (rEO) and left (lEO) external oblique, and right (rPE) and left (lPE) peroneal. A reference electrode was positioned on the right medial malleolus. All procedures were performed by the same experimenter and followed the standards established by SENIAM [33].

**Fig 1:**
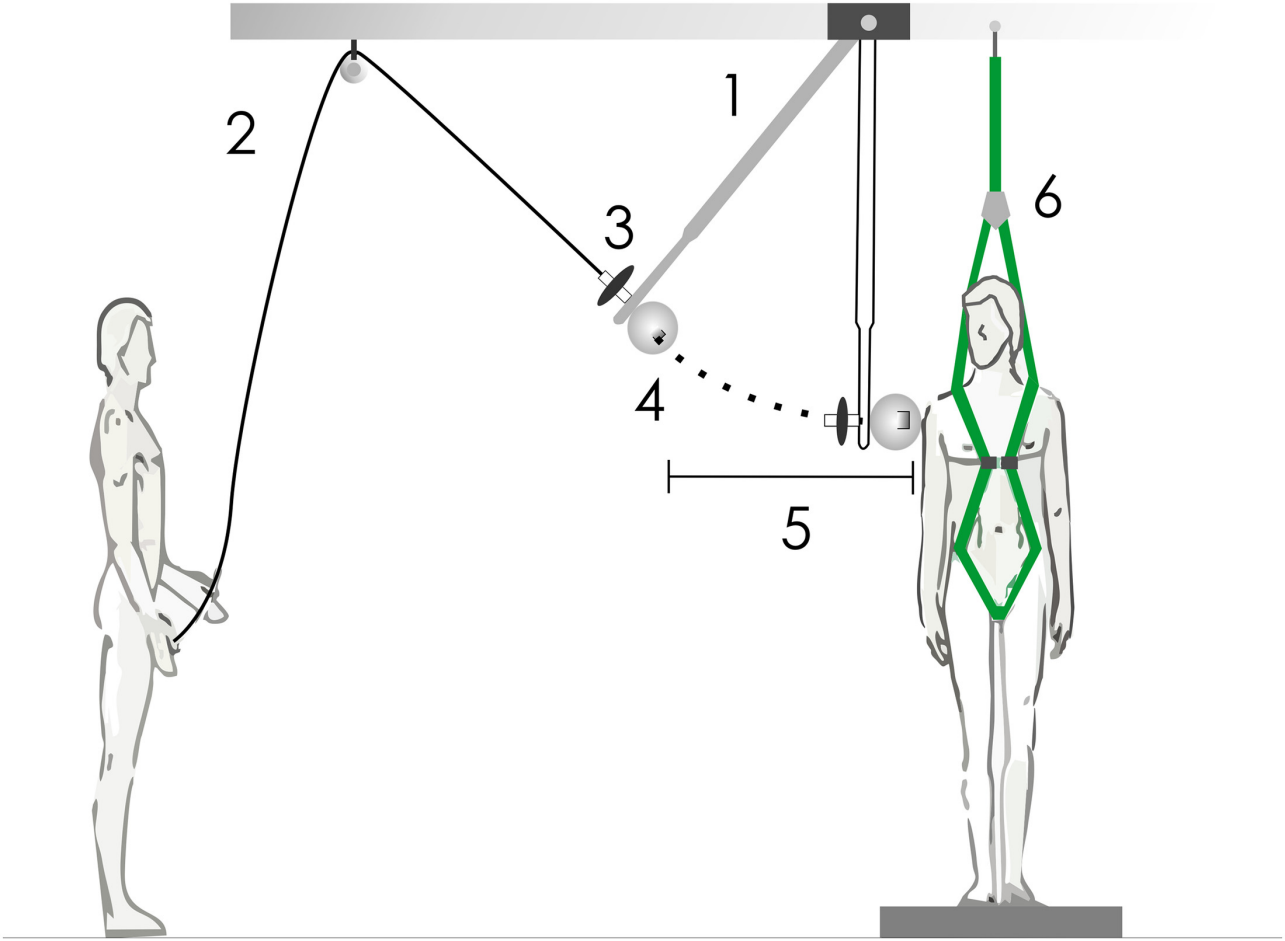
Schematic representation of the pendulum used to induce the postural disturbances. The participants kept their head rotated 30 degrees toward the pendulum. 1: pendulum, 2: pulley system, 3: load attached to the pendulum, 4: ball and the accelerometer, 5: distance of the pendulum release (0.8 m), 6: safety belt.

During data collection, the participants were positioned in a standing position (feet shoulder-width apart) on the force platform by the side of the pendulum. The pendulum consisted of an adjustable (1.0–1.5 meters long) circular (5 cm diameter) aluminum bar, with its proximal end attached to the ceiling of the laboratory and its distal end free for swing. The pendulum distal end was covered with a plastic ball which induced perturbations to the participants’ shoulder. This ball was used for smoothing the impact of the pendulum on the shoulder of the participants. The pendulum was released at a distance of 0.8m from the shoulder of the participants using a pulley system attached to the laboratory ceiling (Fig 1). To increase the magnitude of the postural disturbances, weight plates were attached to the end of the pendulum according to the subjects’ body mass: a 1 kg weight plate was used for participants whose weight was 70 kg or less. A 2 kg weight plate was used for those weighing more than 70 kg. The postural disturbances were induced in the frontal plane (lateromedially) and the participants were instructed to keep their arms relaxed along the sides of the body and keep their head rotated approximately 30 degrees toward the pendulum (between the sagittal and frontal planes). In this way, they were able to see the pendulum trajectory once released by the experimenter (predictable perturbations). They were asked to stop the pendulum with their shoulder, sustaining their posture after the impact. After two attempts to familiarize the participants with the body perturbations, five disturbances were induced on the right side of the participants at the shoulder level. All perturbations were sufficient to generate a feet-in-place postural reaction. Throughout the experiment, subjects used a safety belt to prevent accidents caused by falls.

### Processing and data analysis

To calculate integrals of the muscle activities (∫EMG) during the APA and CPA, the raw data was first rectified and filtered through a digital Butterworth second order low-pass filter (100 Hz). The five individual trials of the smoothed EMG data were seen on the computer screen and aligned according to the first abrupt signal of the accelerometer, i.e., the time of the pendulum impact on the participants’ shoulder. This point was referred to as time zero (t0). The data were cut off 1000ms before t0 and the five trials were then aligned and averaged for each individual. The ∫EMG during the APA and CPA were demarcated between two points (t1 and t2) including four time intervals of 150ms in relation to t0: 1) -250ms to -100ms before t0 to APA1; 2) 100ms before t0 until 50ms after t0 for APA2; 3) 50ms to 200ms after t0 for CPA, and 4) +200ms to +350ms after t0 for CPA2. These time intervals have been widely used in experimental studies that investigate APAs and CPAs in response to postural disturbances [17, 34, 35]. The ∫EMGs were corrected by the ∫EMG from the baseline, which was calculated between -1000 and -950 ms before t0, according to the equation below.

$$\int EMG=\int_{t2}^{t1}EMG-3\int_{-1000}^{-950}EMG$$ (1)

Where ∫*EMG* is the integral of the EMG within the interval determined by the APAs and CPAs ($\int_{t2}^{t1}EMG$) minus 3 times the ∫EMG of the baseline (50ms). In order to compare ∫EMGs between groups, they were normalized as follows: for each subject, the maximal absolute value of a given ∫EMG for a given muscle across all time intervals (APA1, APA2, CPA1, and CPA2) was taken to be unity, and all other values of this particular subject and muscle were divided by this maximal value [10]. The range of ∫EMG index was therefore limited from -1 to 1. Negative and positive values were associated with inhibition and activation of muscle activity.

Postural sway was measured from the displacements of the COP in mediolateral (COPml) and anteroposterior (COPap) directions using the following approximations [36]:

$$COPml=\frac{-(M_{y}+F_{x}*d)}{F_{z}}$$

$$COPap=\frac{M_{x}-F_{y}*d}{F_{z}}$$

Where M_y_ is the moment of force around the frontal axis and M_x_ is the moment of force about the sagittal axis, F_x_ and F_y_ are the horizontal component of the ground reaction force in anteroposterior and mediolateral directions, respectively. F_z_ is the vertical component of ground reaction force and d is the distance between the force platform’s origin and its surface (0.04m).

The average of the COP displacements (COPml and COPap) in the two directions were also calculated between two points, comprising time intervals of 150ms with respect to t0. They were, however, shifted 50ms forward for each time interval to account for the electromechanical delay [37]. This resulted in the following time intervals for COPap and COPml: 1) -200ms to -50ms before t0 for APA1; 2) -50ms before t0 to +100ms after t0 for APA2; 3) +100ms to +250ms after t0 for CPA1; 4) + 250ms to +400ms for CPA2. The positive and negative signs to mediolateral direction represent the left and right sides, respectively. The anterior displacement is a positive signal while the posterior is negative. All data analysis was performed using Matlab (The MathWorks ^®^. Version R2010b, Natick, USA).

### Statistical Analysis

The data obtained with the identification sheet and score through identification questionnaire were analyzed using descriptive statistics (mean, standard deviation, minimum and maximum values). The ∫EMG for each muscle and the COPap and COPml were analyzed using analysis of variance (ANOVA) with mixed model design (2x4). The group was entered as between subject factor and time intervals (APA1, APA2, CPA1 and CPA2) as within subject factor. Tukey post hoc analysis was used to determine differences between the pairs of groups and time intervals with a level of significance of p < 0.05 for each condition.

## Results

### Participants

The averaged data for each group were as follows: PD group: age 63.53 ± 8.32 years old; body mass: 72.84 ± 10.37 kg; UPDRS scale points: 37.69 ± 18.06; modified H & Y scale: 2.33 ± 0.70; time of onset of illness: 7.07 ± 5.10 years. For the control group: age 64.92 ± 8.16 years; body mass: 72.15 ± 7.50 kg (Tables 1 and 2). All the participants were right-side dominant. They were able to follow the task instructions and to generate feet-in-place responses.

**Table 1:** Characteristics of the participants with Parkinson’s disease.

Subjects	Age(yrs.)	Gender	Kg	Height	MMSE	UPDRS H&Y		Time(Yrs.)
1	66	F	60	1.57	26	47	2	7
2	73	M	74	1.60	22	40	2.5	8
3	56	M	79	1.70	28	56	2.5	3
4	44	M	93	1.80	29	28	2	1
5	71	M	70	1.70	29	36	2.5	15
6	65	M	85	1.73	25	39	2.5	10
7	71	F	66	1.63	26	22	1.5	5
8	56	M	74	1.72	28	26	1.5	2
9	61	M	61	1.76	30	29	2.5	6
10	65	F	86	1.59	24	18	1.5	3
11	68	F	58	1.58	25	30	2.5	4
12	61	F	72	1.55	18	85	3	16
13	69	M	69	1.59	28	34	2.5	12
**Mean**	63.53	8/5	72.84	1.65	26	37.69	2.33	7.076

M = male; F = female; Kg = weight in kilograms; MMSE = Mini mental state examination; UPDRS = Unified Parkinson Disease Ratting Scale; H&Y = Modified H&Y.

**Table 2:** Characteristics of the participants in the control group.

		CONTROL			
Subjects	Age(yrs.)	Gender	Kg	Height	MMSE
1	66	F	65	1.65	21
2	74	M	68	1.60	30
3	54	M	68	1.70	28
4	44	M	74	1.74	30
5	71	M	74	1.68	25
6	68	M	80	1.70	25
7	70	F	70	1.56	23
8	66	M	81	1.78	26
9	65	M	70	1.70	30
10	69	F	81	1.56	24
11	65	F	55	1.70	30
12	65	F	80	1.57	26
13	67	M	72	1.63	28
**Mean**	64.92	8/5	72.15	1.66	26.61

M = male; F = female; Kg = weight in kilograms; MMSE = Mini-mental state examination.

### EMG Profile

**Fig 2:**
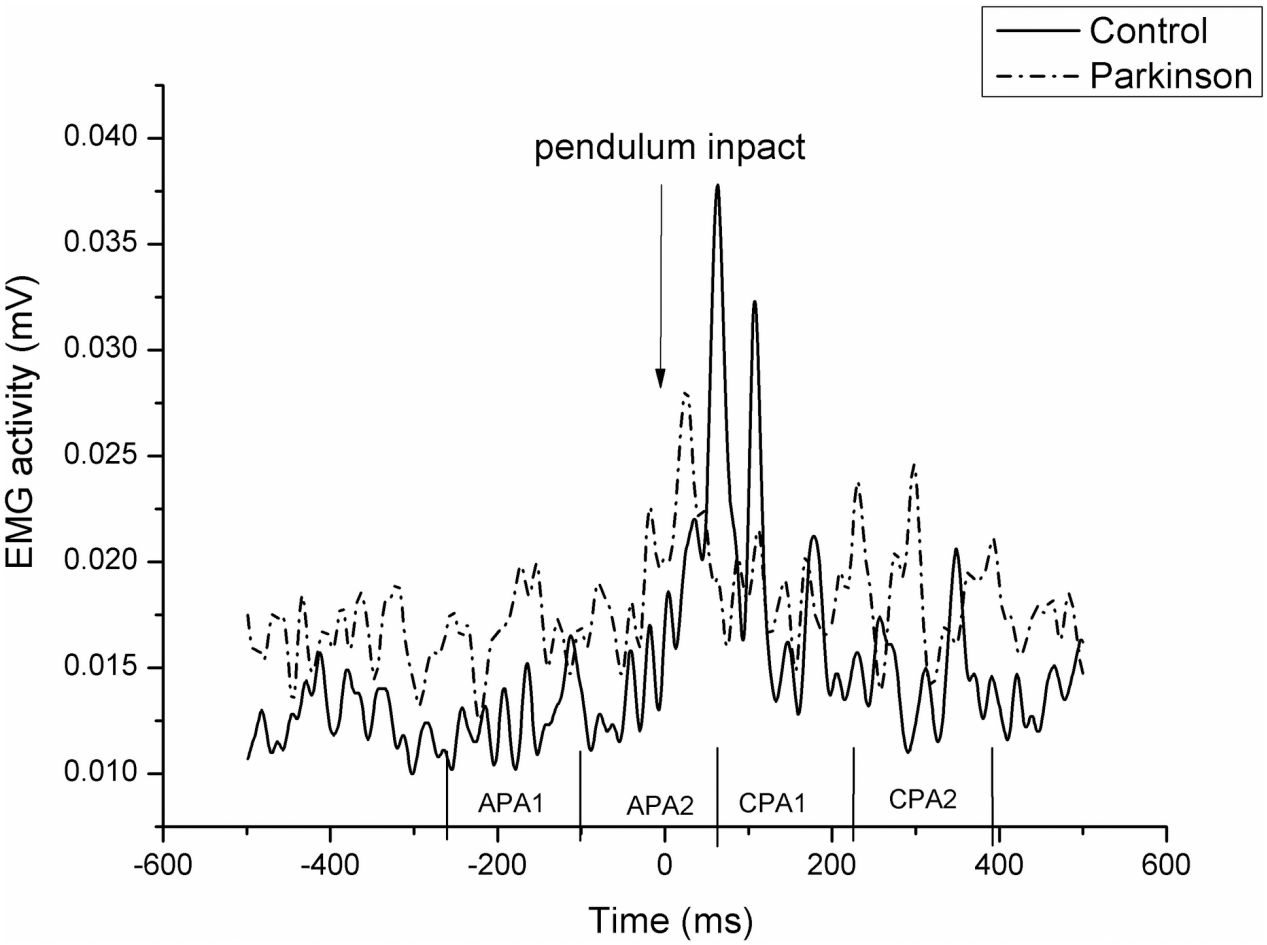
Typical EMG patterns of the right gluteus medius (rGM) activity for two representative subjects (healthy control vs. patient with Parkinson’s disease). The pointing down arrow at time 0 represents the moment of the pendulum impact against the subject’s shoulder.

Fig 2 shows the electrical activity of the rGM muscle for two representative subjects (patient with PD vs. healthy control) during the lateral disturbance. The vertical line represents the disturbance time, i.e., the moment of the pendulum impact (t0) at the participants shoulder. Note that the activity of this muscle was lower in the group with PD, especially after the disturbance (CPA1 time window).

### ∫EMGs

#### Differences between groups

**Fig 3:**
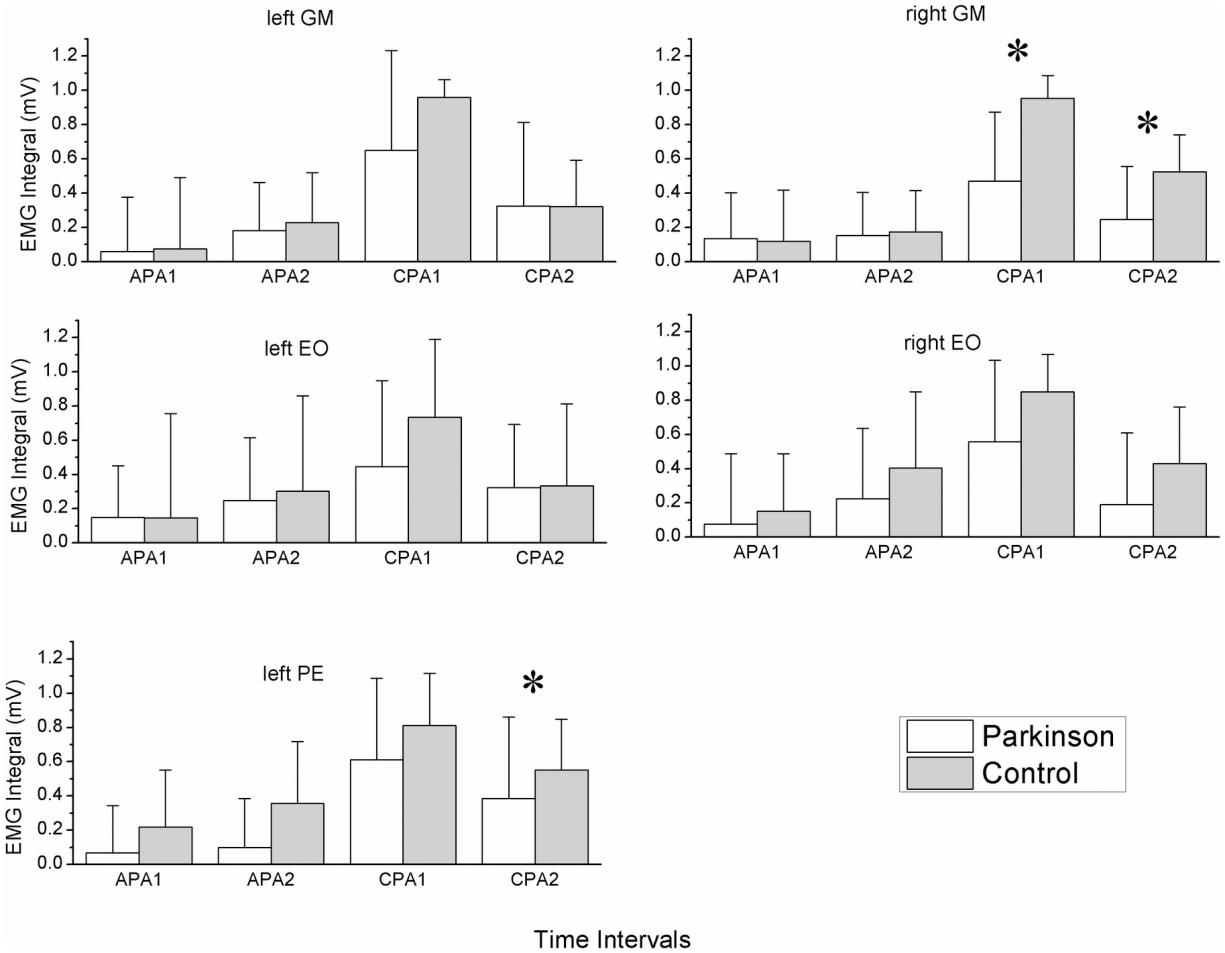
Anticipatory (APA1 and APA2) and compensatory (CPA1 and CPA2) time intervals for the GM, EO and PE right and left lateral muscles for each group (average across 13 subjects). *Significant differences between groups (P<0.05).

The ∫EMGs were statistically significantly lower in the PD group compared to the control group for the following muscles: lPE (F(1, 24) = 6.44 p = 0.018) and rGM (F(1, 24) = 6.60 p = 0.017) (Fig 3). During data processing, we found inconsistences in the rPE EMG signals, which were due to technical problems in the channel of the equipment corresponding to this muscle; hence, the rPE muscle was not used in the statistical analysis.

#### Differences between time intervals

∫EMGs between the time intervals were statistically significant for all muscles (lPE: F(3, 72) = 23.12, p < 0.001; rGM: F(3, 72) = 36.19, p < 0.001; lGM: F(3, 72) = 54.07, p = p < 0.001; rEO: F(3, 72) = 15.47, p < 0.001; lEO: F(3, 72) = 15.63, p < 0.001). The post-hoc analysis showed that, in general, the time interval for CPA1, i.e., just after the impact, was significantly higher than the time intervals for APA1, APA2 and CPA2 in all muscles for both groups (Fig 3).

#### COP displacement

**Fig 4:**
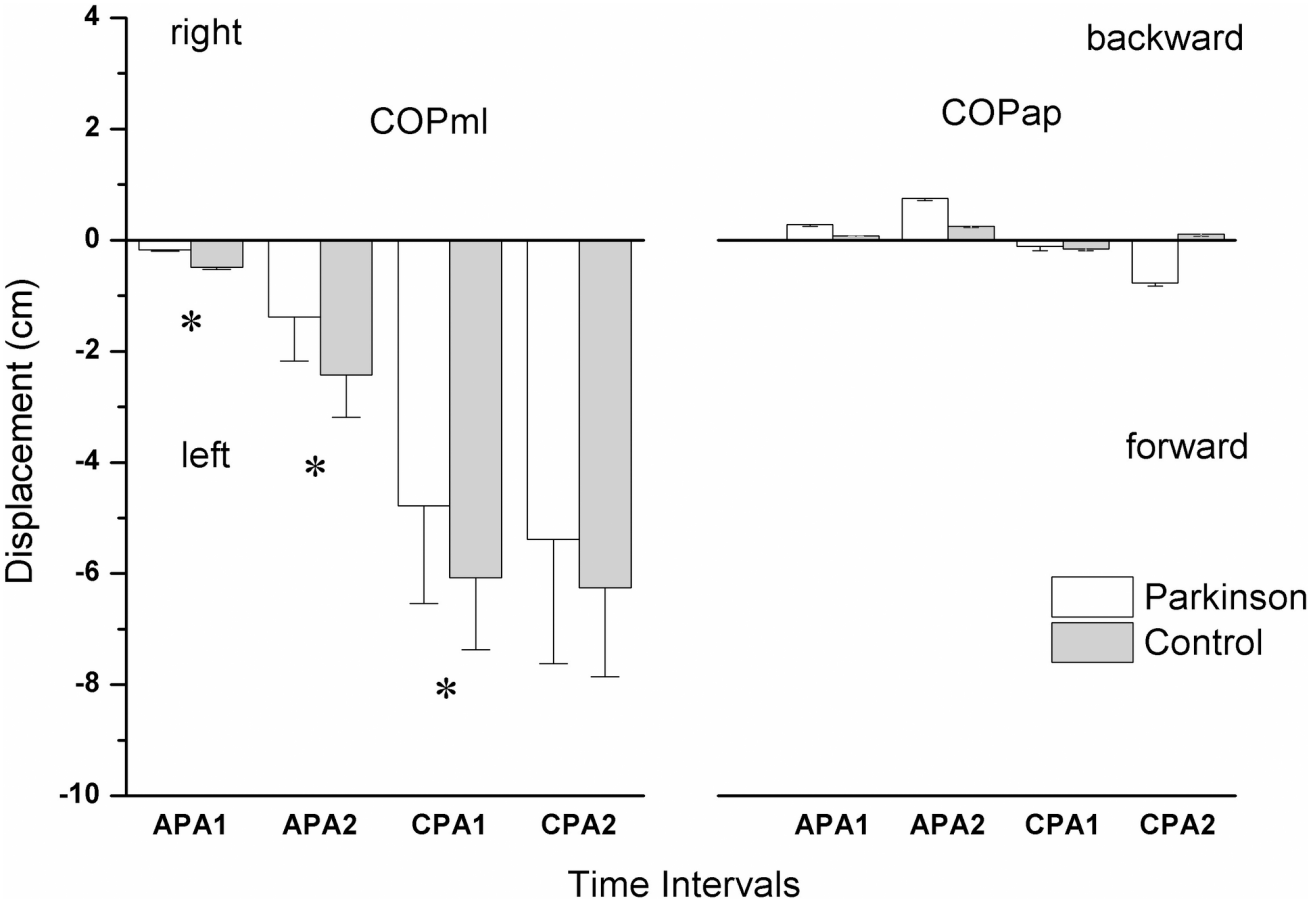
Means and standard errors of the center of pressure displacement in mediolateral (COPml) (right panel) and anteroposterior directions (COPap) (left panel) for the control and PD groups during the 4 time intervals for anticipatory (APA1 and APA2) and compensatory (CPA1 and CPA2) postural adjustments. The positive and negative signs in mediolateral direction represent the left and right sides, respectively. The positive signs denote anterior displacement while negative signs represent posterior excursion.

The COPml displacement was significantly greater in the control than in the PD group (F(1, 24) = 11.575, p <0.001). There was no statistically significant difference between groups in the COPap displacement (F(1, 24) = 0.696, p = 0.406) (Fig 4, right panel). The post hoc analysis detected that the significant differences between groups occurred in the following intervals: APA1 (p<0.001); APA2 (p<0.001) and CPA1 (p<0.001) (Fig 4, left panel). The COPml displacement were statistically significantly different across the time intervals (F(3, 72) = 109, p <0.001) for both groups. In general and in both groups, according to the post-hoc analysis, the COPml displacement during the APA1 and APA2 were significantly lower than the displacement of the COPml during the time intervals CPA1 and CPA2 (all post-hoc p values were below 0.001).

## Discussion

This study investigated the APAs and CPAs in subjects with PD and healthy controls during lateral postural disturbances. We hypothesized that the activity of the postural muscles will be decreased in the group with PD, which will cause a smaller excursion of the COP after the disturbances (CPA). Our hypotheses were partially supported. The subjects with PD showed lower magnitudes of the ∫EMGs only during the CPAs time intervals and not during the anticipatory adjustments (APAs). In addition, they exhibited smaller COP displacements (or postural sway) during both anticipatory and compensatory adjustments as compared to healthy controls. Regardless of these deficits, all subjects with PD were able to attain balance during the predictable lateral perturbations used in this study.

### Differences in ∫EMGs

By inducing predictable external perturbations in the present experiment, we were able to observe a sequence of events in muscular activity, from APAs to CPAs, which participants in both groups used to maintain lateral stability. Both experimental and control groups were able to perceive the perturbations and to produce anticipatory activity in the postural lateral muscles. It has been shown in healthy individuals that, when APAs are available (predictable perturbations), the magnitude of CPAs decreased, while in the absence of APAs, the CPAs are substantially increased [9, 17]. Thus subjects with PD in the present study were able to produce APAs appropriately, but still did not correctly scale (based on controls) the muscular activity and postural sway in the compensatory phase (see below).

Previous studies have shown that the strategies of postural adjustments associated with muscular activity are altered in individuals with PD [18, 20]. Bazalgette and colleagues [18] were the first authors to show APAs deficits in subjects with PD during self-generated perturbations. They reported that the APAs were absent for the most part of subjects with PD during perturbations generated by rapid arm lifting. Later, a study by Latash et al. [10] did not observe differences in APAs between subjects with PD and healthy controls during similar tasks, which is in agreement with the present study. Here, the subjects with PD showed anticipatory muscular activity similar to those observed in the control group. It is possible that the conflicting results between the study by Bazalgette et al. [18] and our own might be related to differences in the experimental tasks. Bazalgette et al. [18] assessed only anteroposterior postural stability induced by self-generated disturbances (internal perturbations), such as fast voluntary movements. This type of perturbation might not be demanding enough to generate important postural perturbations and instability, as subjects with PD are not able to produce faster voluntary movements as compared to healthy subjects [10].

In contrast, the postural disturbances induced by the moving pendulum in our study might have threatened the participants’ postural stability more efficiently than self-generated perturbations. In fact, previous studies showed that APA in subjects with PD depends on the magnitude of motor action. For instance, they showed higher APAs when they were required to release a load, placed in front of them, by fast abduction movement of both shoulders, than when the same load was released by pressing a trigger with their thumb [38]. Subjects with PD might be able to produce appropriate APAs, during tasks with a greater demand for postural control, by over-activating other brain areas such as cortico-cerebellar circuits [39]. In fact, externally-cued urgent tasks (stopping a rolling ball with auditory cue) have shown, via PET scanning, greater activation in cerebellar regions in subjects with PD when compared to healthy controls [40]. However, it is still in debate whether the over-activation of extrastriatal brain regions in subjects with PD is due to compensations or to pathophysiologic changes caused by the disease (see [39] for review).

In addition to the differences in the magnitude of perturbations, the characteristic of the perturbation itself (self-generated vs. predictable external perturbation) might influence APAs [41, 42]. In opposition to a voluntary movement without a goal-directed act (“lift your arm as fast as possible,” i.e., shoulder flexion), the subjects in our study were observing the pendulum/ball trajectory and had a goal-directed act, i.e., “stop the pendulum and maintain your posture/balance.” Altogether, visual and acoustic feedback and a purposeful task (keeping balance) might have enhanced the PD subjects’ performance. Although we did not compare the external lateral perturbations with voluntary movements or unpredictable perturbations, previous studies have shown that subjects with PD are able to improve the motor performance during goal-directed tasks [43] in the presence of acoustic and visual information [40, 44].

Although in our study subjects with PD were able to produce proper APAs, their muscle activities during the CPA phases were altered. Their compensatory muscular activity did not reach the same magnitude of those observed in their matched counterparts. Previous studies involving subjects with PD have shown similar results during unpredictable postural disturbances induced by subtle multidirectional force-platform translations [19]. They showed a decreased magnitude of activity in the tensor fasciae latae muscle along with increased co-activation in these pairs of muscles (left and right) when the disturbances (subtle translations) were generated mediolaterally [19]. In addition to co-activation patterns of the lateral muscles, our results showed decreased muscular activity for the majority of the tested lateral muscles (significant for GM and PE muscles). There are some possible reasons for the observed deficient CPAs. Firstly, changes in dopamine release by the basal ganglia, especially at the caudolateral sensorimotor territories, is associated with poor or loss of automatic and implicit control mechanisms [42]. Secondly, there is growing evidence that Parkinson’s disease impairs proprioception, which adversely influences motor responses [45–47]. For instance, subjects with PD overreacted to vibration stimulation at the Achilles tendon in a standing position, which provokes an illusory forward body movement. Compared to controls, subjects with PD exhibited larger COP displacements into backward direction to compensate for the illusory movements [46, 47]. These results suggest that proprioceptive deficits may affect the ability of subjects with PD to scale postural responses. In fact, subjects with PD exhibited altered compensatory steps during sudden backward platform translations [45]. They showed smaller forward steps when stepping without a visual target than in the presence of it. However, subjects with PD did not increase their step length when the same experiment compared eyes closed and eyes open without visual target conditions. The authors concluded that the observed deficits are likely not related to proprioceptive input to visual input integration; instead, they are related to proprioceptive motor integration centrally mediated. Such a high-order deficiency will prevent subjects with PD to transform proprioceptive input from the peripheral system into an appropriated output in terms of motor response. The results of the aforementioned studies seem contradictory, i.e., hyperactivity to stretch reflex vs. hypometric postural responses (short steps). Altogether, these studies suggested that proprioceptive information might occur in different afferent pathways [46] or are centrally mediated in different locations of the sensorimotor system [45] that controls postural responses. It is possible that the decreased EMG activity after the lateral perturbations found in the present study represent the inability of subjects with PD to transform proprioceptive inputs to adequate CPAs. This is consistent with their difficulty in controlling lateral stability [48] and vulnerability to falls [49].

### Differences in COP displacement

Both groups of participants showed anticipatory and compensatory adjustments in terms of COPml displacements. These displacements ranged from 2 to 6 cm across the time intervals and occurred in the direction opposite to the original direction of the perturbations (negative sign, Fig 4). A similar strategy was observed in past studies when healthy subjects used similar predictable perturbation [11, 16]. It has been suggested that this is a protective strategy to absorb the perturbation impact [50]. In the present study, despite the fact that subjects with PD exhibited proper lateral muscle activity in the APA time intervals, they showed a decrease in COPml excursions during the identical intervals (APA1 and APA2) as compared to the control group (Fig 4, right panel). This, however, might be related to the mechanical changes in the joints and muscles of subjects with PD and not necessarily to the muscular activity. It is extensively known that subjects with PD exhibit increased muscle tone and resistance to joint displacement [51], lower amplitude of trunk lateral flexion [6, 48], axial rigidity [52–54], as well as increased passive stiffness in the leg and pelvis complex [3, 4]. Such a lower flexibility can not only restrict the range of joint movements but also may affect postural control in the presence or absence of disturbances by decreasing postural sway and the excursion towards the limit of stability (LOS) [55, 56]. This rigidity in subjects with PD might have prevented them from swaying in a more effective way to get prepared to absorb the predicted postural perturbation in spite of the “normal” muscular activity at the corresponding time intervals (APA1 and APA2). Such a rigidity, along with proprioceptive deficits, of the subjects with PD, might have also contributed to the observed decreased postural sway (lower COPml displacement) just after the postural disturbance (CPA1). In addition, although we did not calculate the time parameters of the COP displacement in the present study, which is a limitation, there is a possibility that the small displacements of COP were related to COP excursion time. Previous studies showed that the time to the COP peak of excursion after perturbations (movable force platform) is delayed in subjects with PD as compared to healthy participants [56]. Such a “postural bradykinesia” will decrease the magnitude of our calculated COP mean within the time intervals for CPAs (until 400ms). However, time delay responses observed in subjects with PD after force platform translations might not occur during different external perturbations [57, 58]. This fact guarantees future studies involving response time to lateral postural perturbations at the shoulder level in subjects with PD.

It has been proposed that a smaller displacement of COP during static balance represents better postural stability [59–61]. These observations have been based on studies involving healthy individuals [62] and those with neurological [21, 48, 63] and orthopedic disorders [64–66]. However, for the subjects with PD in our study, this finding could not be the case. The magnitude of the lateral perturbations used in the present study make the subjects with PD, and their matched controls, respond to the disturbances using the feet-in-place strategy. Thus the deficient postural responses (CPAs) and lack of flexibility did not prevent the subjects with PD from maintaining their postural equilibrium after the disturbance. However, during more challenging postural perturbations, it is possible that these deficits will prevent these subjects from adjusting their postural sway to absorb body disturbances (perturbation impact), which can be one of the factors that makes subjects with PD prone to falls [49].

## Clinical Implications and Conclusion

The results of this study showed that subjects with PD have reduced magnitude of muscle activity in the CPA time intervals and smaller COPml displacements in the APA and CPA phases during external lateral disturbances of posture. The observed smaller postural sway before and after the body perturbations might be related to proprioceptive impairments and lack of flexibility. These changes may prevent subjects with PD from adjusting postural sway properly and make them more susceptible to falls. The current results may assist in the development of therapeutic interventions aimed to stimulate proprioceptive information and processing to increase muscular activity of lateral postural muscles when facing postural perturbations, especially the gluteus medius. In addition, the lateral flexibility of subjects with PD should be augmented to reach greater joint movements and body excursion in the presence of balance disturbances. Finally, the results of this study can motivate new studies to investigate therapeutic interventions that optimize the use of postural adjustment strategies in subjects with PD.
